# Wistin Exerts an Anti-Inflammatory Effect via Nuclear Factor-κB and p38 Signaling Pathways in Lipopolysaccharide-Stimulated RAW264.7 Cells

**DOI:** 10.3390/molecules27175719

**Published:** 2022-09-05

**Authors:** Jangeun An, Gyoungah Ryu, Seong-Ah Shin, Huiji Kim, Mi-Jeong Ahn, Jun Hyuck Lee, Chang Sup Lee

**Affiliations:** 1 College of Pharmacy and Research Institute of Pharmaceutical Sciences, Gyeongsang National University, Jinju 52828, Korea; 2 Research Unit of Cryogenic Novel Material, Korea Polar Research Institute, Incheon 21990, Korea; 3 Department of Polar Sciences, University of Science and Technology, Incheon 21990, Korea

**Keywords:** inflammation, phytochemical, wistin

## Abstract

Inflammation is an immune response to cellular damage caused by various stimuli (internal or external) and is essential to human health. However, excessive inflammatory responses may be detrimental to the host. Considering that the existing drugs for the treatment of inflammatory diseases have various side effects, such as allergic reactions, stomach ulcers, and cardiovascular problems, there is a need for research on new anti-inflammatory agents with low toxicity and fewer side effects. As 4′,6-dimethoxyisoflavone-7-*O*-β-d-glucopyranoside (wistin) is a phytochemical that belongs to an isoflavonoid family, we investigated whether wistin could potentially serve as a novel anti-inflammatory agent. In this study, we found that wistin significantly reduced the production of nitric oxide and intracellular reactive oxygen species in lipopolysaccharide-stimulated RAW 264.7 cells. Moreover, wistin reduced the mRNA levels of pro-inflammatory enzymes (inducible nitric oxide synthase (iNOS) and cyclooxygenase (COX-2)) and cytokines (interleukin (IL)-1β and IL-6) and significantly reduced the protein expression of pro-inflammatory enzymes (iNOS and COX-2). Furthermore, wistin reduced the activation of the nuclear factor-κB and p38 signaling pathways. Together, these results suggest that wistin is a prospective candidate for the development of anti-inflammatory drugs.

## 1. Introduction

Inflammation is the body’s immune response and defense mechanism against tissue damage caused by exposure to harmful or toxic external agents, infections, and physical injuries [1]. Inflammation resulting from infection or injury is associated with inflammatory responses such as immune cell recruitment and accumulation, release of inflammatory mediators, and changes in blood vessel permeability [2]. Generally, there are two types of inflammation: acute and chronic. Acute inflammation is a rapid process that repairs quickly to minimize damage and restore tissue homeostasis [3]. However, in chronic conditions, the inflammatory response continues, resulting in severe organ damage [4]. Chronic inflammation promotes the progression of several diseases, including cardiovascular disease, inflammatory bowel disease, rheumatoid arthritis and diabetes [5]. Crohn’s disease (CD) is the most prevalent IBD syndrome treated using 6-mercaptopurine (6-MP) and its prodrug azathioprine (AZA) [6]. As immunosuppressive drugs, 6-MP and AZA inhibit inflammation by blocking the body’s immune response; however, they may cause disease recurrence and side effects, such as hepatotoxicity [6]. Sulfasalazine, a medication for patients with rheumatoid arthritis, has immunomodulatory properties and suppresses pro-inflammatory cytokines; however, it can cause gastrointestinal side effects, such as headache, dizziness, rash, and bone marrow suppression [7]. Non-steroidal anti-inflammatory drugs (NSAIDs) are anti-inflammatory drugs used worldwide to treat inflammatory conditions [8]. Among NSAIDs, ibuprofen (IBU) is a non-selective inhibitor of both cyclooxygenase (COX)-1 and -2 isozymes [9]; however, it is associated with the risk of cardiovascular and gastrointestinal complications [10,11]. Naproxen is another NSAID used to treat osteoarthritis, migraine, and rheumatoid arthritis [10]. However, NSAIDs have several side effects, including gastrointestinal toxicity, cardiovascular risk, kidney damage, and hepatotoxicity [8]. Therefore, it is necessary to develop safer anti-inflammatory treatment strategies.

When the human body is infected by pathogens in damaged tissue, immune cells recognize pathogen-associated molecular patterns (PAMPs) and damage-associated molecular patterns (DAMPs) using pattern recognition receptors (PRRs) and promote inflammatory signaling pathways [12]. The Toll-like receptor (TLR) family is mainly expressed in immune cells as major PRRs that play key roles in the induction of innate immune responses and first-line defense [13,14]. Lipopolysaccharide (LPS) is a well-known inflammatory PAMP molecule that exists in the cell walls of gram-negative bacteria [13]. The TLR4–LPS interaction activates the nuclear factor-κB (NF-κB) and mitogen-activated protein kinase (MAPK) pathways [15,16]. TLR4-mediated modulation of myeloid differentiation factor 88 (MyD88) results in activation of NF-κB. Furthermore, NF-κB is regulated by the IκB and IκB kinase (IKK) complex [17], which causes IκB phosphorylation. The phosphorylation of the IκB protein results in the phosphorylation of p65, which translocates to the nucleus [18]. In addition, NF-κB is regulated by phosphatidylinositol 3-kinase (PI3K)-Akt [19]. The MAPK pathway includes extracellular signal-regulated kinase (ERK1/2), c-Jun N-terminal kinase (JNK), and p38, which result in further activation of the transcription factor activator protein 1 (AP-1) [16]. Activation of the NF-κB and MAPK pathways has been reported to increase the expression of inflammatory enzymes (inducible nitric oxide synthase (iNOS) and cyclooxygenase (COX-2)) and cytokines (interleukin (IL)-1β and IL-6) in immune cells [16,20]. Therefore, understanding the mechanism by which signaling pathways are controlled at the molecular level might help modulate the inflammatory response and develop new modulators of inflammation.

Phytochemicals extracted from plants have emerged as new agents for the treatment of chronic inflammatory diseases [21]. Current anti-inflammatory disease treatments (non-steroidal anti-inflammatory drugs and glucocorticoids) have many side effects, including tissue damage, cardiovascular problems, and liver complications. As plant-derived drugs have been reported to have fewer toxic effects, it has been suggested that they could be potential modulators of inflammation [22,23]. Human papillomavirus (HPV) infection can cause chronic inflammation due to the release of pro-inflammatory cytokines [24,25]. Sinecatechin extracted from green tea has been used as a medication to treat HPV infections [24,25]. Silymarin extracted from the seeds of milk thistle (*Silybum marianum*) has anti-inflammatory activity; it inhibits NF-κB activity and improves liver function in patients with hepatitis B (HBV) [26,27,28]. Eupatilin, a plant-derived drug extracted from *Artemisia asiatica Nakai*, is used to treat gastritis by mediating anti-inflammatory effects and promoting the regeneration of offended mucosa [29]. Among various isoflavones, genistein is known for its antioxidant and anti-inflammatory properties. Genistein prevents endothelial inflammatory damage by inhibiting the NF-κB pathway, which mediates the transcription of proinflammatory cytokines [30]. Although investigations are required to confirm its safety and efficacy, low- (5–15 mg/kg/day) and high-dose (160 mg/kg/day) genistein treatments for patients with mucopolysaccharidosis (MPS) III reportedly have no major side effects [31]. Another isoflavone, daidzein, inhibits the JNK, PARP, and NF-κB signaling pathways to express pro-inflammatory cytokines [32]. In addition, plant polyphenols, particularly flavonoids, have exhibited anti-inflammatory activity both in vitro and in vivo [32]. Compound 4’,6-dimethoxyisooflavone-7-*O*-β-d-glucopyranoside (wistin), belonging to the isoflavone family, is an agonist of PPARγ and PPARα in adipocytes and hepatocytes, respectively [33,34]. However, its anti-inflammatory role has not been investigated. Therefore, in this study, we aimed to identify its anti-inflammatory effects and elucidate the molecular mechanisms underlying its anti-inflammatory effects in LPS-stimulated RAW264.7 cells.

## 2. Results

### 2.1. Effects of Wistin on Cell Viability in LPS-Induced RAW 264.7 Cells

**Figure 1:**
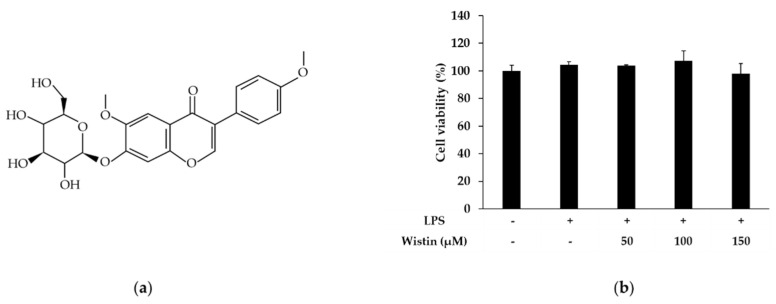
The effects of wistin on cell viability in LPS-induced RAW 264.7. (**a**) The chemical structure of wistin. (**b**) The cells were treated with the indicated concentrations of wistin (0, 50, 100, 150 µM) for 30 min prior to treatment with LPS (0.1 µg/mL) for 24 h, and then cell viability was examined using MTT assay. The data are presented as the means ± SD; *n* = 3.

Because isoflavones have been reported to be cytotoxic at high doses, we attempted to confirm whether wistin is cytotoxic at high concentrations [35]. The cytotoxicity of wistin (Figure 1a) on the viability of RAW 264.7 cells was evaluated using the 3-(4, 5-dimethylthiazolyl-2)-2, 5-diphenyltetrazolium bromide) (MTT) assay. Cotreatment of wistin (50 μM, 100 μM, and 150 μM) and LPS (0.1 μg/mL) did not show cytotoxicity in RAW 264.7 at 24 h (Figure 1b).

### 2.2. Effects of Wistin on the Production of Pro-Inflammatory Mediators in LPS-Induced RAW 264.7 Cells

**Figure 2:**
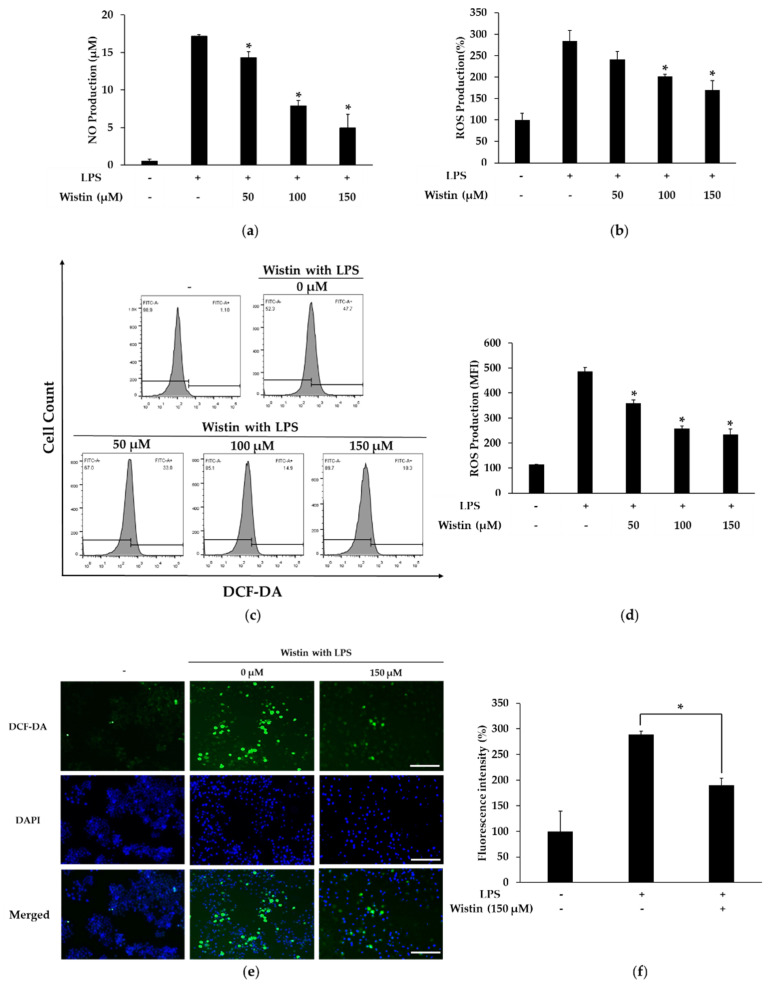
The effect of wistin on NO and ROS production in LPS-stimulated RAW264.7. The cells were treated with the indicated concentrations of wistin for 30 min prior to treatment with LPS (0.1 µg/mL) for 24 h. (**a**) The NO production was measured in treated wistin in the culture supernatant. ROS production was measured on a microplate reader (**b**), FACS (**c**,**d**), and fluorescence microscope (**e**). (**f**) Quantitative analysis of ROS production using ImageJ software. The scale bar represents 100 μm. * *p* < 0.05 compared with the LPS-treated group. MFI: mean fluorescence intensity. The data are presented as the means ± SD; *n* = 3.

Prolonged inflammatory processes increase the production of nitric oxide (NO) and reactive oxygen species (ROS), leading to tissue dysfunction [36]. ROS can regulate pro-inflammatory gene expression, and NO is an important pro-inflammatory mediator in inflammatory signaling [37]. To examine the anti-inflammatory effects of wistin, we investigated the production of NO and ROS. Wistin showed a significant decrease in LPS-induced NO production in a dose-dependent manner compared to that in the control group (Figure 2a). In addition, dose-dependent inhibition of intracellular ROS generation by wistin was identified using a microplate reader, fluorescence-activated cell sorting (FACS), and fluorescence microscopy (Figure 2b–e). These data suggest that wistin can reduce the production of pro-inflammatory mediators (NO and ROS) induced by LPS.

### 2.3. Effects of Wistin on Pro-Inflammatory Enzymes and Cytokine Gene Expression in LPS-Induced RAW 264.7 Cells

**Figure 3:**
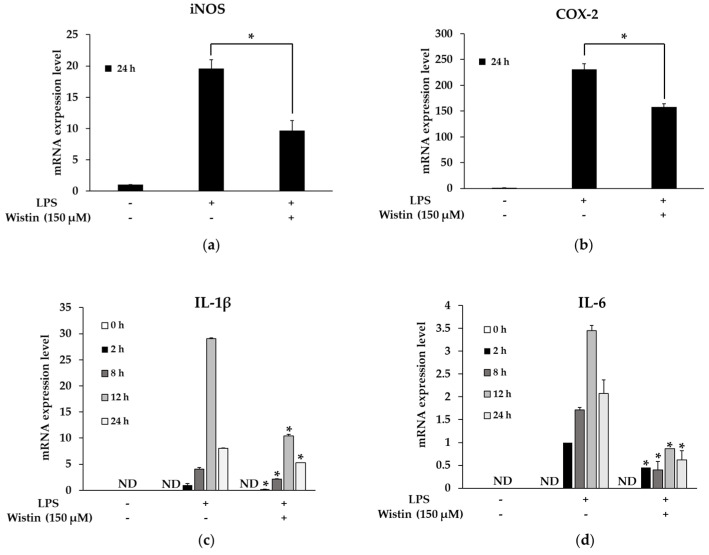
The effects of wistin on mRNA expression level in LPS-induced RAW 264.7 cells. The cells were treated with the indicated concentrations of wistin for 30 min prior to treatment with LPS (0.1 µg/mL). Then, LPS-induced (**a**) iNOS, (**b**) COX-2, (**c**) IL-1β, and (**d**) IL-6 mRNA level were measured using a quantitative reverse transcription polymerase chain reaction (qRT-PCR). * *p* < 0.05 compared with the LPS-treated group. ND: not detected. The data are presented as the means ± SD; *n* = 3.

Next, we investigated the involvement of wistin in the modulation of mRNA levels of inflammatory enzymes (iNOS and COX-2) and pro-inflammatory cytokines (IL-1β and IL-6) at indicated time points after LPS treatment [38]. Wistin significantly decreased the mRNA expression levels of inflammatory enzymes (iNOS and COX-2) (Figure 3a,b). The expression levels of pro-inflammatory cytokines (IL-1β and IL-6) were not detected at 0 h after LPS treatment and were the highest at 12 h after LPS treatment. In addition, wistin-treated groups showed a significant decrease in the mRNA expression levels of pro-inflammatory cytokines at each time point compared to LPS treatment groups. Therefore, these results suggest that wistin could modulate pro-inflammatory enzymes and cytokine gene expression induced by LPS.

### 2.4. Effects of Wistin on the Protein Expression Level of Pro-Inflammatory Enzymes in LPS-Induced RAW 264.7 Cells

**Figure 4:**
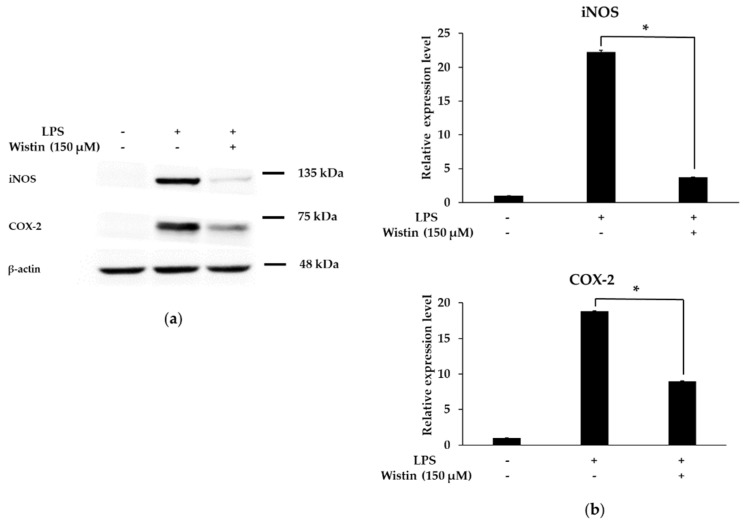
The effects of wistin on protein expression of iNOS and COX-2 in LPS-induced RAW 264.7 cells. The cells were treated with the indicated concentrations of wistin for 30 min prior to treatment with LPS (0.1 µg/mL) for 24 h. (**a**) The protein levels of pro-inflammatory enzymes iNOS and COX-2 were determined by Western blot. (**b**) Quantitative analysis of the iNOS/β-actin and COX-2/β-actin using image J. * *p* < 0.05 compared with the LPS-treated group. The data are presented as the means ± SD; *n* = 2.

The expression of iNOS and COX-2 regulates key inflammatory mediators [39]. Therefore, we investigated whether wistin exhibits anti-inflammatory effects by inhibiting iNOS and COX-2 protein expression. As shown in Figure 4a,b, wistin significantly reduced the protein expression levels of iNOS and COX-2 compared to those in the LPS group. Therefore, these results suggest that wistin could also inhibit the expression of pro-inflammatory enzymes at the protein level.

### 2.5. Effects of Wistin on the Activation of AKT/NF-κB Pathway in LPS-Induced RAW 264.7 Cells

**Figure 5:**
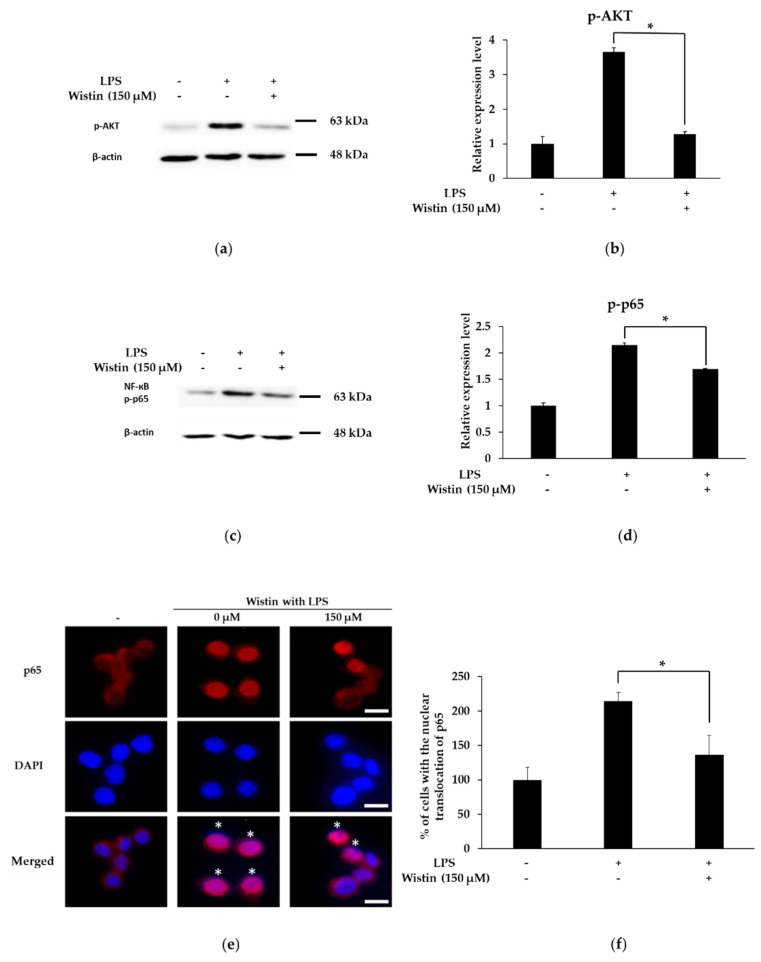
The effects of wistin on p-AKT, NF-κB (p-p65 subunit) in LPS-induced RAW 264.7 cells. The cells were treated with the indicated concentrations of wistin for 30 min prior to treatment with LPS (0.1 µg/mL) for 2 h. Then, the phosphorylation of AKT (**a**) and p65 (**c**) were measured using a Western blot. Quantitative analysis of the p-AKT/β-actin (**b**) and p-p65/β-actin (**d**) using image J. (**e**) The effects of wistin on the nuclear translocation of p65 (red) using fluorescence microscopy. The merged images were acquired by overlaying two channels (p65 (red) and DAPI (blue)). * Indicates translocation of p65 from the cytoplasm to the nucleus. The scale bar represents 10 μm. (**f**) Quantitative analysis of the nuclear translocation of p65 using ImageJ software. * *p* < 0.05 compared with the LPS-treated group. The data are presented as the means ± SD; *n* = 3.

The NF-κB pathway regulates the expression and production of pro-inflammatory enzymes and cytokines [40]. Therefore, we examined the phosphorylation level of LPS-induced AKT and NF-κB (p65 subunit) following wistin (150 µM) treatment for 2 h. Wistin significantly reduced the phosphorylation level of AKT and p65 compared to that in the LPS group (Figure 5a–d). Furthermore, in the absence of LPS stimulation, p65 (red in the merged image) was present in the cytoplasm. Upon LPS stimulation, p65 (pink in merged images) was translocated to the nucleus, whereas wistin treatment reduced the nuclear translocation of p65 (red and pink in merged images) (Figure 5e). These results suggest that wistin suppresses the AKT/NF-κB signaling pathway.

### 2.6. Effects of Wistin on the Activation of MAPK Pathway in LPS-Induced RAW 264.7 Cells

**Figure 6:**
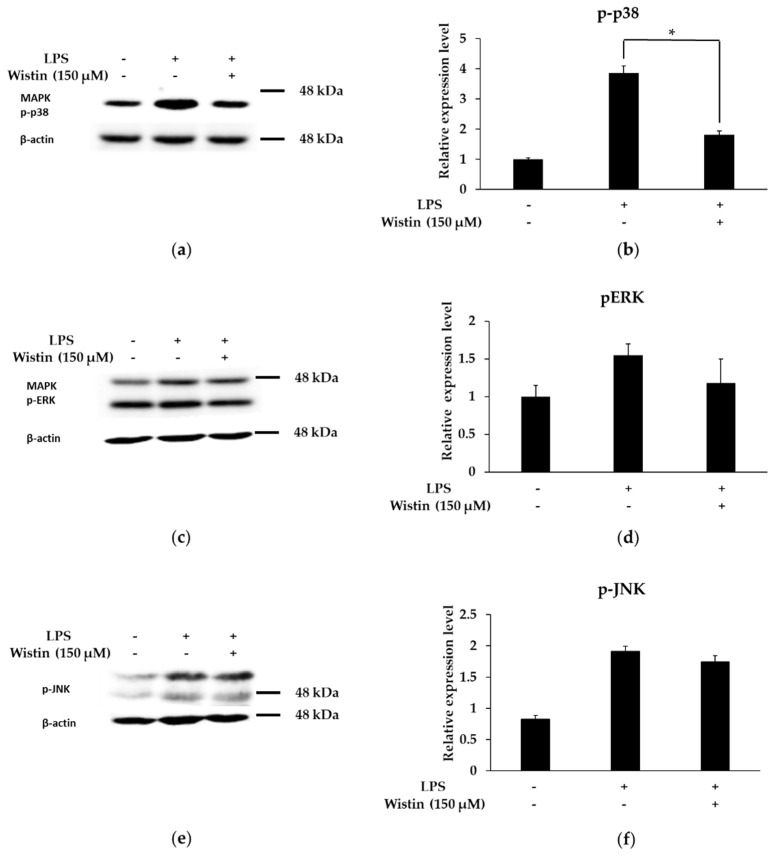
The effects of wistin on MAPK (p-p38, p-ERK, and p-JNK) in LPS-induced RAW 264.7cells. The cells were treated with the indicated concentrations of wistin for 30 min prior to treatment with LPS (0.1 µg/mL) for 2 h. Phosphorylation of p38 (**a**), ERK, (**c**) and JNK (**e**) was measured using a Western blot. Quantitative analysis of the p-p38/β-actin (**b**), p-ERK/β-actin (**d**), and p-JNK/β-actin (**f**) using image J. * *p* < 0.05 compared with the LPS-treated group. The data are presented as the means ± SD; *n* = 2.

The MAPK pathway is known for its role in the modulation of inflammatory responses [41]. We investigated the role of wistin in the phosphorylation of three MAPKs (p38, c-Jun N-terminal kinase (JNK), and extracellular signal-regulated kinase (ERK)). Phosphorylation of p38 was decreased by wistin, but that of ERK and JNK was not affected compared to that in the LPS group (Figure 6). Therefore, these results suggest that wistin could work by negatively regulating the p38 MAPK pathway.

## 3. Discussion

Inflammation is a protective response to harmful stimuli (PAMP and DAMP) by the immune system [42]. However, chronic inflammation can cause various diseases, such as cardiovascular disease, inflammatory bowel disease, rheumatoid arthritis, and diabetes [5]. Ibuprofen, naproxen, and other NSAIDs are conventional anti-inflammatory drugs used to treat rheumatism, osteoarthritis, arteriosclerosis, neuroinflammatory diseases, and other inflammatory diseases [8,10]. However, these drugs have several side effects, such as an increased risk of gastric mucosal injury, renal injury, and other medical complications [8,10]. Therefore, it is important to identify novel anti-inflammatory agents that can overcome the shortcomings of conventional anti-inflammatory drugs. Several plants have been used as folk medicines for the treatment and prevention of diseases [43]. Currently, sinecatechin extracted from green tea and silymarin extracted from milk thistle seeds (*Silybum marianum*) are used as plant-derived anti-inflammatory drugs for treating HPV and HBV infections, respectively [24,25,26,27]. Furthermore, as plant-derived anti-inflammatory drugs, eupatilin and JOINS tablets are used to treat gastritis and knee osteoarthritis [44,45]. Isoflavones have antioxidant, anticancer, antibacterial, and anti-inflammatory properties [32]. Our study highlights the potential of wistin as an anti-inflammatory agent with fewer side effects via modulation of the inflammatory signaling pathway. However, further in vivo and clinical studies of wistin in this regard are warranted.

During inflammation, NO plays an important role in the regulation of immune and inflammatory responses [46]. Additionally, the high production of ROS during inflammation can lead to cell damage through the oxidation of DNA, RNA, and proteins [47]. iNOS and COX-2 are pro-inflammatory mediators that are regulated by pro-inflammatory transcription factors [48]. When we examined the effects of wistin on NO and ROS generation, we found that wistin reduced the levels of both NO and ROS. In addition, wistin significantly decreased the mRNA expression of inflammatory enzymes (iNOS and COX-2) and inflammatory cytokines (IL-1β and IL-6). Furthermore, wistin decreased the protein expression of iNOS and COX-2. Therefore, these results suggest that wistin exerts anti-inflammatory effects by modulating inflammatory enzymes, inflammatory mediators, and cytokines.

NF-κB signaling is a well-known inflammatory pathway that regulates the expression of pro-inflammatory cytokines (IL-1β and IL-6) and pro-inflammatory enzymes (iNOS and COX-2) [49]. p65 (RelA) is a component of NF-κB, and phosphorylation of p65 induces the expression of a variety of genes [50]. In addition, AKT regulates NF-κB by phosphorylating the IκB kinase (IKK) complex, which phosphorylates the p65 subunit [19]. Wistin reduced the phosphorylation of AKT and p65 in LPS-stimulated RAW264.7. In addition, wistin decreased the translocation of p65 from the cytosol to the nucleus. These results suggest that wistin exerts anti-inflammatory effects by inhibiting the AKT/NF-κBsignaling pathway.

The MAPK pathway (ERK, JNK, and p38) regulates pro-inflammatory mediators [41]. In particular, p38 can induce NF-κB activation to induce the expression of pro-inflammatory cytokines [51]. p38 MAPK activates mitogen- and stress-activated protein kinases (MSK), which can activate NF-κB (p65 subunit) signaling [52]. Wistin decreased LPS-induced p38 phosphorylation but has no effect on LPS-induced EKR and JNK phosphorylation. These results suggest that wistin could have anti-inflammatory effects by inhibiting p38 pathways in LPS-stimulated RAW264.7.

**Figure 7:**
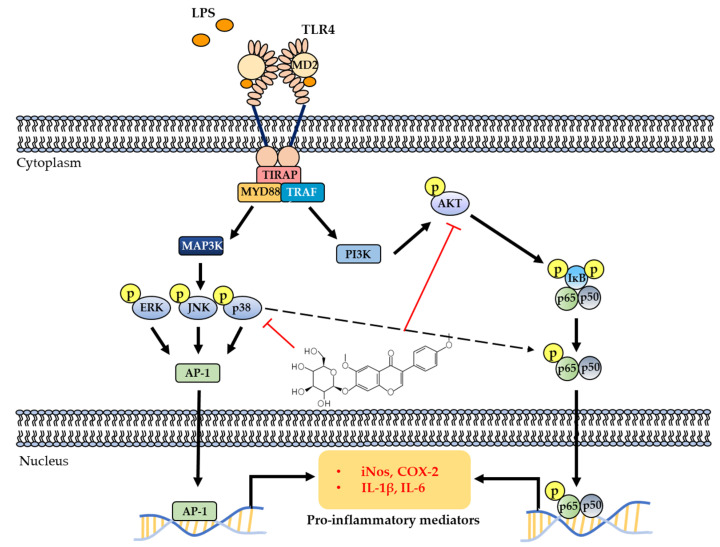
Wistin suppressed LPS-stimulated inflammation by inhibiting the phosphorylation of the p65 and p38 signaling pathways.

In conclusion, we demonstrated that wistin exerts anti-inflammatory effects by downregulating pro-inflammatory mediators in LPS-mediated signaling. Wistin could regulate pro-inflammatory mediators, including NO, ROS, pro-inflammatory cytokines, and enzymes, by inhibiting the NF-κB and p38 signaling pathways (Figure 7). Similarly, genistein and daidzein inhibit inflammatory mediators via the NF-κB and MAPK signaling pathways [30,32]. They belong to the isoflavonoid family and exhibit versatile pharmacological activities [30,32]. Therefore, wistin may be developed as a plant-derived anti-inflammatory agent with fewer side effects than other conventional anti-inflammatory drugs.

## 4. Materials and Methods

### 4.1. Reagents

Wistin was purchased from Indofine Chemical Company, Inc. (Hillsborough, NJ, USA) and was dissolved in dimethyl sulfoxide (DMSO). Dulbecco’s modified Eagle’s medium (DMEM, high glucose) was purchased from Hyclone Laboratories Inc. (Marlborough, MA, USA), and fetal bovine serum (FBS) was purchased from Corning (Corning, NY, USA). Penicillin-streptomycin-glutamine was purchased from Gibco (Waltham, MA, USA). MTT, LPS from *Escherichia coli* O127:B8, N-(1-naphtyhyl) ethylenediamine dihydrochloride, phosphoric acid, sulfanilic acid, and nitrite ion standard solutions were purchased from Sigma-Aldrich Co. (St. Louis, MO, USA). Dulbecco’s phosphate-buffered saline (DPBS) and Hank’s balanced salt solution (HBSS) were purchased from WELGENE, Inc. (Gyeongsan, Korea). 2′7-Dicholrodihydrofluorecsein diacetate (DCF-DA) was purchased from Cayman Chemical Company (Ann Arbor, MI, USA). The quantitative reverse transcription polymerase chain reaction (qRT-PCR) primers were purchased from Macrogen (Seoul, Korea). Primary antibodies ((iNOS (#13120), COX-2(#12282), phospho-p65 (#13346), phospho-JNK (#9255), phospho-ERK (#4370), and β-actin (#8457)) were purchased from Cell Signaling Technology, Inc. (Danvers, MA, USA). Goat anti-rabbit IgG (5220-0036) and goat anti-mouse IgG (5220-0341) antibodies were purchased from SeraCare Life Sciences, Inc. (Gaithersburg, MD, USA).

### 4.2. Cell Culture

The RAW264.7 cell line (mouse origin) was obtained from Dr. Sung Ho Ryu’s lab (POSTECH, Korea). The cells were cultured in DMEM supplemented with 10% FBS and 1% penicillin–streptomycin and incubated at 37 °C with 5% CO_2_.

### 4.3. Cell Viability

RAW 264.7 cells were plated in a 48-well plate at a density of 3 × 10^4^ cells/well. After 24 h, RAW 264.7 cells were pre-treated with 50, 100, and 150 µM of wistin for 30 min, followed by LPS (0.1 µg/mL) treatment and incubation for 24 h. After that, the DMEM medium was removed, and 1mg/mL of MTT solution was added to each well and was further incubated for 2 h at 37 °C. The solution was removed from the wells and the formed formazan crystals were dissolved in 200 µL of DMSO. Finally, absorbance was measured at 570 nm using a Varioskan LUX Multimode Microplate Reader (Thermo Fisher Scientific Co., Waltham, MA, USA).

### 4.4. Measurement of NO Production

The Griess test was used to determine NO production. RAW 264.7 cells were plated into a 24-well plate at a density of 6 × 10^4^ cells/well. After 24 h, RAW 264.7 cells were pre-treated with 50, 100, and 150 µM of wistin for 30 min, followed by treatment with LPS (0.1 µg/mL) and further incubation for 24 h. The supernatant from each well was transferred to a 96-well plate, followed by the addition of Griess reagent in a 1:1 ratio. After 15 min, absorbance was measured at 540 nm using a microplate reader (Varioskan LUX Multimode Microplate Reader, Thermo Fisher Scientific Co.).

### 4.5. Measurement of ROS Production

ROS production was determined using the 2’,7’-dichlorofluorescin-diacetate (DCF-DA) assay. RAW 264.7 cells were plated into a 24-well plate at a density of 6 × 10^4^ cells/well. After 24 h, RAW264.7 cells were pre-treated with 50, 100, and 150 µM wistin for 30 min, followed by LPS (0.1 µg/mL) treatment and further incubation for 24 h. The solution was completely removed from all the wells before incubation with a DCF-DA probe for 30 min at 37 °C. The cells were washed three times with HBSS before measuring fluorescence. The fluorescence intensity was measured at excitation and emission wavelengths of 485 nm and 530 nm, respectively, using a microplate reader (Varioskan LUX Multimode Microplate Reader, Thermo Fisher Scientific Co.). ROS in intact cells were detected with fluorescence microscopy using a Zeiss microscope (Zeiss, Jena, Germany). ROS production was further analyzed using flow cytometry (BD FACSVerse™, BD Bioscience).

### 4.6. QRT-PCR

RAW264.7 cells plated into a 6-well plate at a density of 3 × 10^5^ cells/well were pre-treated with 150 µM of wistin for 30 min, followed by LPS (0.1 µg/mL) treatment. After 0, 2, 8, 12, and 24 h, cells were washed with DPBS and harvested. Total RNA was isolated using the TRIzol reagent (Invitrogen, Waltham, MA, USA). cDNA was synthesized from total RNA using a SimpliAmp Thermal Cycler (Applied Biosystems Co., Waltham, MA, USA), and then qRT-PCR was performed on a Step One Plus Real-time PCR System Cycler (Applied Biosystems Co.) using a Power SYBR Green PCR mix Cycler (Applied Biosystems Co.). The PCR primer sequences (forward and reverse) used are listed in Table 1, and β-actin was used as the reference gene.

### 4.7. Western Blot Analysis

Protein isolation was performed using RAW264.7 cells plated in a 60 mm culture dish at a density of 7 × 10^5^ cells/well. After 24 h, RAW264.7 cells were pre-treated with 150 µM wistin for 30 min, followed by LPS (0.1 µg/mL) treatment. The protein was isolated at two time points, 2 and 24 h. Cells were harvested and lysed with lysis buffer (10 mM Tris pH 7.4, 150 mM NaCl, 1 mM EDTA pH 8, 1% Triton X-100, 1% sodium deoxycholate, 30 mM NaF, 1.5 mM NaVO4, 1 mM PMSF, and 1 mg/mL each of aprotinin, leupeptin, and pepstatin A) and sonicated for 10 s. The cell lysates were centrifuged at 13,000 rpm for 10 min at 4 °C (Centrifuge 5424 R (rotor type: FA-45-30-11), Eppendorf, Hamburg, Germany) and quantified with the help of the Bradford (Abcam, Cambridge, UK) assay. The proteins (15 µg) were subjected to sodium dodecyl sulfate–polyacrylamide gel electrophoresis and transferred to a nitrocellulose membrane. The membranes were blocked with 5% skim milk in Tris-buffered saline supplemented with 0.1% Tween-20 (TBS-T) at room temperature (RT) for 30 min. Membranes were then incubated with suitable primary antibodies overnight at RT with gentle shaking. The following day, the membranes were washed with TBS-T for 30 min. Membranes were then incubated with secondary horseradish peroxidase (HRP)-conjugated anti-rabbit/mouse IgG antibodies for 1 h at RT. Finally, the membranes were detected using an ECL reagent (Cytiva, Malborough, MS, USA), and the bands were visualized using Chemidoc (iBright™ CL1500, Invitrogen). Band intensities were analyzed using ImageJ software. β-actin was used as the loading control.

### 4.8. Immunofluorescence

RAW264.7 cells plated into a 4-well plate with a density of 6 × 10^4^ cells/well were pre-treated with 150 µM of wistin for 30 min, followed by LPS (0.1 µg/mL) treatment. After 2 h, cells were washed with PBS and fixed with 4% paraformaldehyde (Sigma-Aldrich; Merck KGaA) in PBS for 20 min. The cells were then incubated with 0.2% Triton X-100 and 0.1% citrate (Sigma-Aldrich; Merck KGaA) in PBS for 5 min, washed with PBS, and blocked with 2% bovine serum albumin (Sigma-Aldrich; Merck KGaA) for 30 min, followed by probing with rabbit anti-p65 NF-κB antibody (1:500; cat. no. 8242; Cell Signaling Technology, Inc.) for 2 h. After washing with 2% bovine serum albumin, the cells were incubated with fluorescein goat anti-rabbit IgG (H+L) cross-adsorbed secondary antibody Alexa Fluor 555 (4 µg/mL; cat. no. A-21428; Invitrogen Inc., Middlesex County, MA, USA) for 1 h. After washing with 2% bovine serum albumin, the nuclei were counterstained with DAPI solution (1 mg/mL) for 5 min in the dark, and fluorescence was visualized using a fluorescence microscope (Zeiss microscope, Carl Zeiss AC).

### 4.9. Statistical Analysis

Results are presented as mean ± standard deviation (SD). Two statistical analysis methods were used. ANOVA and Dunnett’s post hoc tests were conducted to compare three or more groups. The Student’s *t*-test was conducted to compare two groups. Statistical significance was set at *p* < 0.05(*).

## Figures and Tables

**Table 1:** Primers used in the quantitative reverse transcription polymerase chain reaction.

Name of the Primer	Primer Sequence
*iNos*	Forward 5′-GAACGGAGAACGTTGGATTTG-3′
	Reverse 5′-TCAGGTCACTTTGGTAGGATTT-3′
*Cox-2*	Forward 5′-GAAGATTCCCTCCGGTGTTT-3′
	Reverse 5′-CCCTTCTCACTGGCTTATGTAG-3′
*Tnf-α*	Forward 5′-ACGTGGAACTGGCAGAAGAG-3′
	Reverse 5′-GGTCTGGGCCATAGAACTGA-3′
*Il-6*	Forward 5′-TCTGAAGGACTCTGGCTTTG-3′
	Reverse 5′-GATGGATGCTACCAAACTGGA-3′
*Il-1β*	Forward 5′-AGGTCAAAGGTTTGGAAGCA-3′
	Reverse 5′-TGAAGCAGCTATGGCAACTG-3′
*β-actin*	Forward 5′-ATGGAGGGGAATACAGCCC-3′
	Reverse 5′-TTCTTTGCAGCTCCTTCGTT-3′

## Data Availability

Not applicable.
